# A bibliometric review of 35 years of studies about preeclampsia

**DOI:** 10.3389/fphys.2023.1110399

**Published:** 2023-02-02

**Authors:** Razieh Akbari, Sedigheh Hantoushzadeh, Zahra Panahi, Sajedeh Bahonar, Marjan Ghaemi

**Affiliations:** ^1^ School of Medicine, Department of Obstetrics and Gynecology, Tehran University of Medical Sciences, Tehran, Iran; ^2^ University of Tehran, Tehran, Iran

**Keywords:** bibliometric, preeclampsia, web of science database, co-citation, co-occurrence, theme evolution

## Abstract

The purpose of this study is to investigate preeclampsia. It used the visualization tools of CiteSpace, VOSviewer, Gunnmap, Bibliometrix^®^, and Carrot2 to analyze 3,754 preeclampsia studies from 1985 to 2020 in Obstetrics and Gynecology areas. Carrot2 was used to explain each cluster in extra detail. The results found that there is an increasing trend in many publications related to preeclampsia from 1985 to 2020. The number of studies on preeclampsia has increased significantly in the last century. Analysis of the keywords found a strong relationship with preeclampsia concepts and keywords classified into five categories. Co-citation analysis was also performed which was classified into six categories. Reading the article offers important to support not only to grind the context of preeclampsia challenges but also to design a new trend in this field. The number of studies on preeclampsia has substantially improved over the decades ago. The findings of documents published from 1985 to 2020 showed three stages in research on this subject: 1985 to 1997 (a seeding stage), 1997–2005 (rapid growth stage), and 2005 onwards (development stage).

## Introduction

As one of the multisystem diseases during pregnancy, pre-eclampsia is a considerable cause of maternal and infant morbidity and mortality worldwide (Steegers et al., 2010). Over the past century, the term pre-eclampsia has evolved from a kidney-specific disease causing chronic nephritis to a state of toxaemia caused by circulating toxins (Phipps et al., 2016), characterized by hypertension and proteinuria appearing after 20 weeks of gestation. Pre-eclampsia affects approximately 5–7% of pregnancies (Witlin & Sibai, 1998). Recently, it has been redefined as *de novo* hypertension that manifests after 20 weeks of pregnancy and is accompanied by proteinuria (N300 mg/day), maternal organ dysfunction (including renal insufficiency, liver involvement, neurological or haematological problems), or uteroplacental dysfunction (potential cause of fetal growth restriction) (Tranquilli et al., 2014).

Bibliometric or citation analysis evaluates frequently cited articles in various disciplines, reviews the redundancy of the literature, assesses the quality of publications, highlights trends in research interest, and/or the evolution of scholarly publications over time. It is widely used in different medical fields and several studies using this approach have been published (Lee et al., 2019; Yadava et al., 2019), such as traumatic brain injury (Karydakis et al., 2019), artificial intelligence in the treatment of cerebrovascular disease and heart disease (Tran et al., 2019), IUGR (Kazemi Aski et al., 2020), and oral leukoplakia (Liu W et al., 2019).

To the best of our knowledge, no bibliometric research on preeclampsia has been performed. This study was therefore designed as a bibliometric analysis to evaluate pre-eclampsia articles from 1985 to 2020. To examine the data present in the WoS database, a quantitative literature search was employed. The relationships and impacts of key publications and related factors in this field were assessed using literature metrics, article ratings, and institutional and country ratings.

Our article makes several contributions to the advancement of literature. To uncover thinking flows, essential concepts, and developing issues with the potential to be included in future studies, we first utilized the quantitative bibliometric analysis method, which enables many numerous robust structured, and thorough inspections of this research subject. Subsequently, in order to demonstrate how the preeclampsia domain’s bounds have changed over time and give readers an immediate understanding of the deftly constructed increasing. Finally, we connected evolutionary paths with upcoming study directives to promote new preeclampsia study flows in the Obstetrics and Gynecology field.

## Methods

### Bibliometrics method

Bibliometrics is a systematic method that is broadly defined as a quantitative examination of published documents (Broadus, 1987; De Bellis, 2009). Using approaches such as content analysis, text analysis, citation analysis, keyword co-occurrence, co-citation analysis, or co-authoring analysis, Pritchard described bibliometrics as the application of mathematics and statistical methodologies to books and other media of communication (Pritchard, 1969; Dias, 2019). By highlighting the most significant articles, the bibliometric analysis might help the scholar avoid becoming overwhelmed by the massive amount of publications (Karydakis et al., 2019). Utilizing citation data, citation analysis quantifies the study’s influence as a reflection of the volume of citations a publication accumulates over time (Yadava et al., 2019). Additionally, citation analysis is employed to assess academic impacts (Liu et al., 2013). The papers with a high citation count are assumed to contain insightful suggestions for further studies (Jeung et al., 2011) and their authors are regarded as significant figures in the field (Butt et al., 2019).

The majority of bibliometric studies used citation analysis to determine the most popular papers, authors, journals, countries, co-authorship of authors, and keyword co-occurrences (Alarcon-Ruiz et al., 2019; Lee et al., 2019). Citation analysis has been applied in a variety of fields to identify the most significant publications (States et al., 2019). The frequency with which a text has been cited by other researchers can be determined using techniques of citation analysis (Xu et al., 2015). Citation analysis methods have recently made their way into the medical studies (Chen, 2004; Gupta et al., 2019).

### Eligibility requirements, study selection, and data extraction

The study had nothing to do with the approval of ethical committees. The WoS database was used to retrieve the data for our study. Data were gathered in December 2020 utilizing the WoS database’s internet search engine. In this study, the authors conducted a comprehensive search of all papers using the term “Preeclampsia” as the search query. Only the “TITLE” was the subject of keyword searches. As a result, a group of 3754 papers written between 1985 and 2020 were gathered. Then, this collection was fixed as the foundation for all ensuing VOSviewer analyses. The application CiteSpace and VOSviewer (1.6.11) was used to create bibliometric network visualizations (Chen, 2004; Van Eck & Waltman, 2009).

**FIGURE 1:**
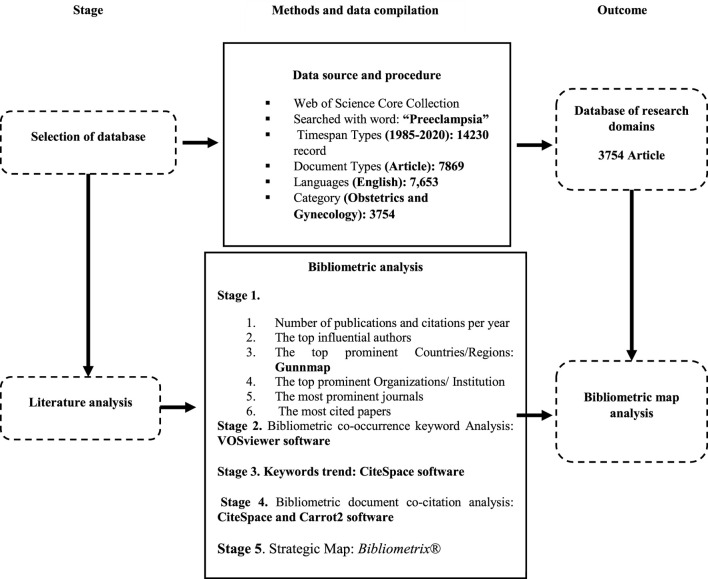
Methodological approach (Author’s Presentation).

A review of the literature revealed that 14,230 publications about preeclampsia were published between 1985 and 2020. Article (3,754), Proceedings Paper (221), Early Access (62), and book chapter are among these publications (13). In this study, 7,653 of the 7,869 English-language papers that fall under the category of obstetrics and gynaecology were examined. The total number of articles eventually decreased to 3,754 (Figure 1).

### Data synthesis

Country maps were created using Gunnmap. VOSviewer software was used to examine the bibliometric co-occurrence keyword analysis, CiteSpace for the analysis of keywords trends, Carrot2 for the analysis of bibliometric document co-citations, and Bibliometrix^®^ for the analysis of the strategic map.

## Results

### Distribution of published preeclampsia papers per year

**FIGURE 2:**
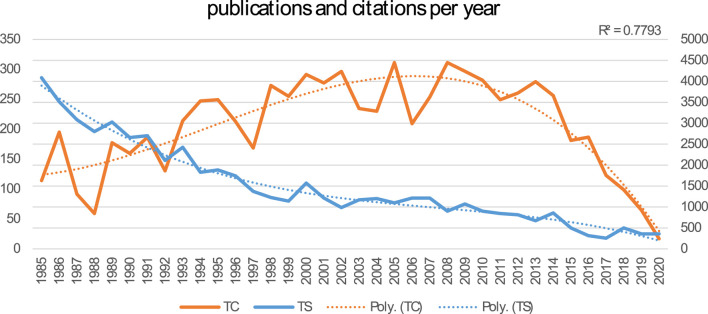
Number of publications and citations per year from 1985 to 2020.

A total of 3,495 papers were included. Figure 2 shows the growth in preeclampsia research from 1985 to 2020 in terms of publications (total studies/TS) and citations (total citations/TC) each year. Over half of the articles (51% of them) were published within the last 10 years.

### Authors with the most publications in the preeclampsia discipline

The top 20 document-producing authors, nations/regions, and organizations/institutions are recognized. The ranked authors who reproduced preeclampsia articles between 1985 and 2020 were Sibai, B.M. (TS = 98); Roberts, J.M. (TS = 64); Romero, R. (TS = 55); Nicolaides, K.H. (TS = 48); Martin, JN (TS = 32); Chaiworapongsa, T (TS = 30); Hassan, S.S. (TS = 29); Steegers EAP (TS = 28); Baker PN (TS = 27); Saade GR (TS = 27); Staff, A.C. (TS = 28); Dekker, G.A. (TS = 26); Erez O (TS = 26); Van Pampus MG (TS = 26); Nisell H (TS = 25); Karumanchi, S.A. (TS = 24); Redman CWG (TS = 24); Von Dadelszen P (TS = 24); Wang YP (TS = 24); and Franx A (TS = 22).

### Countries/regions with the most publications in the preeclampsia discipline

**FIGURE 3:**
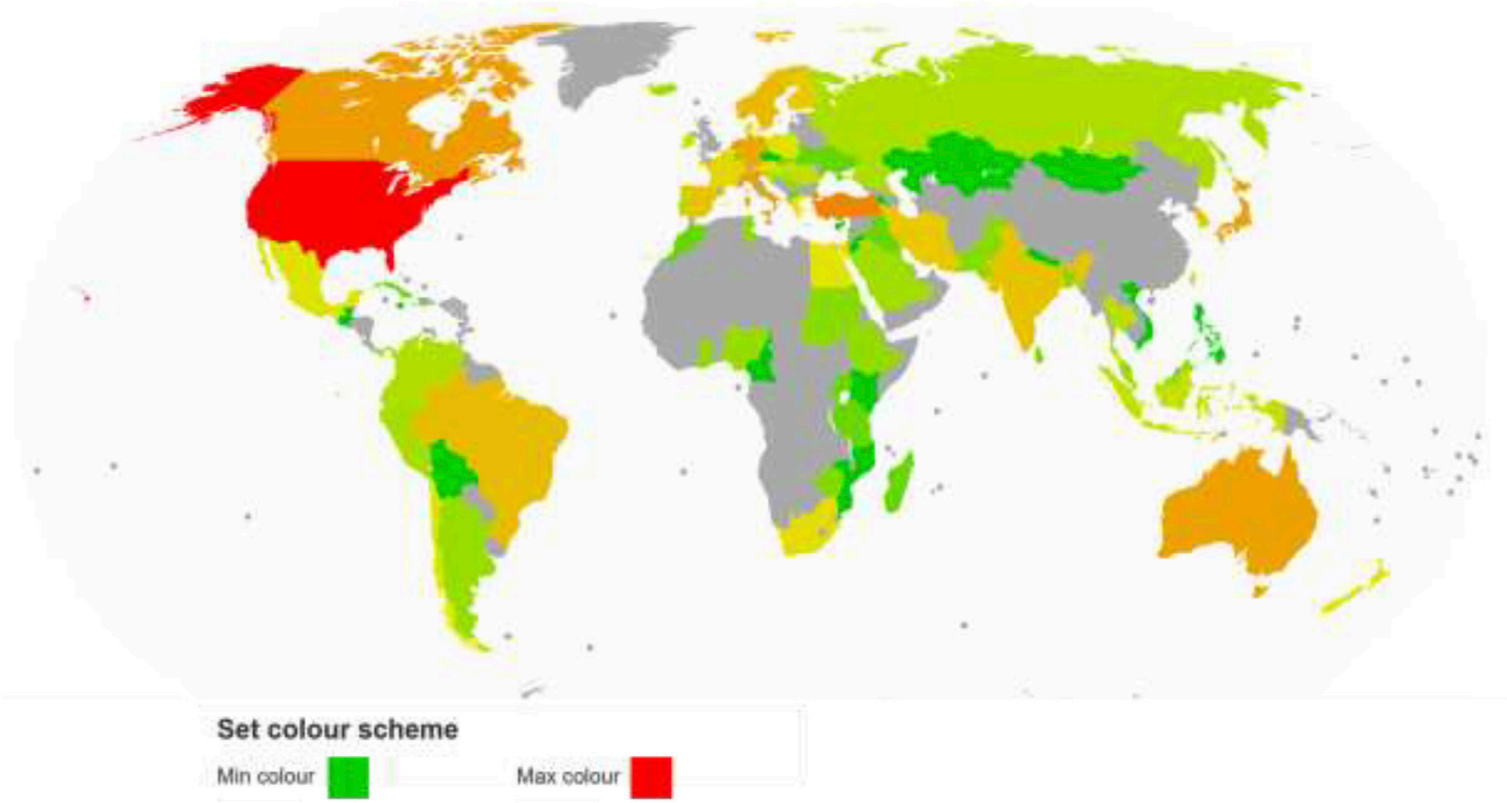
Regions map based on documents.

The number of articles generated by each region and the total number of citations each country has on the examined study area define the impact of the most productive regions. We select the top 20 document-producing areas in the preeclampsia field of research. The “United States” is the first dominant region (TS = 1200, TC = 49,692), and after that “Peoples R China” (TS = 311, TC = 3307) and “England” (TS = 262, TC = 12,342). Another region with a high number of publications is Turkey (TS = 232), followed by the Netherlands (TS = 196), Canada (TS = 162), Italy (TS = 152), Japan (TS = 149), Australia (TS = 145), Israel (TS = 131), Germany (TS = 130), Norway (TS = 101), Sweden (TS = 93), Brazil (TS = 91), India (TS = 80), Spain (TS = 79), South Korea (TS = 72), Iran (TS = Figure 3 displays a map of the study-based regions in red (highest) and green (lowest) colours.

### Organizations/institutions with the most publications in the preeclampsia discipline

The study includes 2359 institutions from various geographical areas. The top 20 organisations with the most publications are listed below. The impact of the most productive organisations in the preeclampsia discipline is determined by the total number of documents printed by each organisation and the total number of citations. The three most significant universities are the “University of Texas system” (TS = 108, TC = 3845), “Harvard University” (TS = 104, TC = 4124), and “The Pennsylvania Commonwealth System of Higher Education/PCSHE” (TS = 99, TC = 5616). Other institutions with the highest publications include, ‘The University of London’ (TS = 97, TC = 97), ‘The University of Pittsburgh’ (TS = 95, TC = 5463); ‘NIH USA’ (TS = 92, TC = 5949), ‘The University of Tennessee system’ (TS = 86, TC = 6219), ‘University of Tennessee health science center’ (TS = 84, TC = 6150), ‘NIH Eunice Kennedy Shriver national institute of child health human development NICHD’ (TS = 79, TC = 5448), ‘University of California system’ (TS = 78, TC = 4319); ‘Wayne State University’ (TS = 76, TC = 4518); ‘Magee Women’s research institute’ (TS = 69, TC = 4082), and ‘Kings College London’ (TS = 67, TC = 3426), ‘University of Oslo’ (TS = 59, TC = 2117); ‘Karolinska Institutet’ (TS = 57, TC = 1320), and ‘Erasmus university Rotterdam’ (TS = 54, TC = 1248); ‘The University of Cincinnati’ (TS = 52, TC = 3118), ‘The University of Groningen’ (TS = 52, TC = 1361), ‘King S College Hospital’ (TS = 51, TC = 2435), and ‘King S college hospital NHS foundation trust’ (TS = 51, TC = 2435).

### Journals with the most publications in the preeclampsia discipline

This section introduces the 20 top Journals in order. The ‘AJOG’ is the most influential journal, with 41,991 total citations (TS = 622). The ‘Hypertension in Pregnancy’ (7247 citations, TS = 447), the ‘Journal of Maternal Fetal and Neonatal Medicine’ (3911 citations, TS = 297), the ‘Pregnancy hypertension an international journal of Womens cardiovascular health’ (1691 citations, TS = 280), the ‘Obstetrics and Gynecology’ (15,156 citations, TS = 267), the ‘Placenta’ (6038 citations, TS = 209), the ‘European journal of obstetrics gynecology and reproductive biology’ (3599 citations, TS = 152), the ‘Acta Obstetricia et Gynecologica Scandinavica’ (2763 citations, TS = 113), the ‘Reproductive sciences’ (1988 citations, TS = 113), the ‘Gynecologic and obstetric investigation’ (1948 citations, TS = 109), the ‘Archives of gynecology and obstetrics’ (1077 citations, TS = 88), the ‘American Journal of Perinatology’ (1029 citations, TS = 82), the ‘BMC Pregnancy and Childbirth’ (826 citations, TS = 79), the ‘Journal of Perinatal Medicine’ (1295 citations, TS = 79), the ‘Prenatal diagnosis’ (1377 citations, TS = 52), the ‘Clinical and Experimental Obstetrics Gynecology’ (277 citations, TS = 47), the ‘Fetal diagnosis and therapy’ (1159 citations, TS = 44), the ‘Journal of Reproductive Medicine’ (451 citations, TS = 43), the ‘British Journal of Obstetrics and Gynaecology’ (BJOJ) (3928 citations, TS = 41), the ‘Journal of the society for gynecologic investigation’ (1048 citations, TS = 40).

### Most-cited documents in preeclampsia discipline

The most cited documents involved Roberts et al. (1989) (N = 1466); Khong et al. (1986) (N = 1247); Sacks et al. (1998) (N = 607) and Walsh (1985) (N = 546) respectively (Table 1). Also, the results found that 24 (out of 30 most cited) of the documents were clinical and research papers and 6 of the documents were literature reviews. About 18 articles were published in ‘AJOG’. Table 2.

**TABLE 1:** The most cited authors, countries, organizations, and journals in the preeclampsia discipline.

	Authors	TS	TC	Countries/Regions	TS	TC	Organizations/Institution	TS	TC	Journals	TS	TC
1	SibaiB.M.	98	7420	United States of America	1200	49,708	University of Texas System	108	3845	AJOG	622	41,991
2	RobertsJ.M.	64	5824	Peoples R China	311	3311	Harvard University	104	4124	Hypertension in Pregnancy	447	7247
3	Romero, R	55	3900	England	262	12,347	PCSHE	99	5616	Journal of Maternal-Fetal and Neonatal Medicine	297	3911
4	NicolaidesK.H.	48	2154	Turkey	232	3163	University of London	97	4191	Pregnancy hypertension an international journal of Women’s cardiovascular health	280	1691
5	Martin, JN	32	1414	Netherlands	196	6583	University of Pittsburgh	95	5463	Obstetrics and Gynecology	267	15,156
6	Chaiworapongsa, T	30	2108	Canada	162	5922	NIH United States of America	92	5949	Placenta	209	6038
7	HassanS.S.	29	1612	Italy	152	3712	University of Tennessee system	86	6219	European journal of obstetrics gynaecology and reproductive biology	152	3599
8	Steegers EAP	28	701	Japan	149	3545	University of Tennessee health science center	84	6150	Acta Obstetricia et Gynecologica Scandinavica	113	2763
9	Baker PN	27	990	Australia	145	3019	NIH Eunice Kennedy Shriver national institute of child health human development NICHD	79	5448	Reproductive sciences	113	1988
10	Saade GR	27	1052	Israel	131	3490	University of California system	78	4319	Gynecologic and obstetric investigation	109	1948
11	StaffA.C.	27	1087	Germany	130	3148	Wayne State University	76	4518	Archives of gynaecology and obstetrics	88	1077
12	DekkerG.A.	26	1719	Norway	101	4276	MAGEE Womens research institute	69	4082	American Journal of Perinatology	82	1029
13	Erez O	26	1167	Sweden	93	2715	Kings College London	67	3426	BMC Pregnancy and Childbirth	79	826
14	Van Pampus MG	26	548	Brazil	91	1148	University of Oslo	59	2117	Journal of Perinatal Medicine	79	1295
15	Nisell H	25	622	India	80	696	Karolinska Institutet	57	1320	Prenatal diagnosis	52	1377
16	KarumanchiS.A.	24	1254	Spain	79	1631	ERASMUS University Rotterdam	54	1248	Clinical and Experimental Obstetrics Gynecology	47	277
17	Redman CWG	24	1838	South Korea	72	1708	University of Cincinnati	52	3118	Fetal diagnosis and therapy	44	1159
18	Von Dadelszen P	24	1401	Iran	69	491	University of Groningen	52	1361	Journal of Reproductive Medicine	43	451
19	Wang YP	24	1149	Finland	65	2423	King S College Hospital	51	2435	British Journal of Obstetrics and Gynaecology	41	3928
20	Franx A	22	632	France	53	967	King S College hospital NHS foundation trust	51	2435	Journal of the society for gynecologic investigation	40	1048

**TABLE 2:** The top studies within the preeclampsia discipline.

References	Source	Citation	Citation per year	Research type
Roberts et al. (1989)	AJOG	1466	44.42	Literature review
Khong et al. (1986)	BJOJ	1247	34.64	Literature review
Sacks et al. (1998)	AJOG	607	25.29	Clinical
Walsh (1985)	AJOG	546	14.76	Clinical
Gilstrap & Ramin (2002)	Obstetrics and Gynecology	534	26.7	Clinical
von Dadelszen et al. (2003)	Hypertension in Pregnancy	475	25	Literature review
Romero et al. (2008)	Journal of Maternal-Fetal & Neonatal Medicine	454	32.43	Clinical
Sibai et al. (1986b)	AJOG	445	12.36	Clinical
Roberts & Hubel (2009)	Placenta	441	33.92	Literature review
Dekker, et al. (1995)	AJOG	424	15.7	Clinical
MacKay et al. (2001)	Obstetrics and Gynecology	414	19.71	Clinical
Kurki et al. (2000)	Obstetrics and Gynecology	390	17.73	Clinical
Ness & Roberts (1996)	AJOG	382	14.69	Literature review
Ishihara et al. (2002)	AJOG	380	19	Clinical
Sibai et al. (1995)	AJOG	362	13.41	Clinical
Sibai et al. (1997)	AJOG	361	14.44	Clinical
Saftlas et al. (1990)	AJOG	358	11.19	Clinical
Lisonkova & Joseph (2013)	AJOG	347	38.56	Clinical
Vince et al. (1995)	BJOJ	338	12.52	Clinical
Yallampalli & Garfield (1993)	AJOG	330	11.38	Clinical
Pineles et al. (2007)	AJOG	322	21.47	Clinical
Allaire et al. (2000)	Obstetrics and Gynecology	320	14.55	Clinical
Seligman et al. (1994)	AJOG	314	11.21	Clinical
Villar et al. (2006)	AJOG	307	19.19	Clinical
Sibai et al. (1994)	AJOG	301	10.75	Clinical
Akolekar et al. (2013)	Fetal Diagnosis and Therapy	287	31.89	Clinical
Ødegård et al. (2000)	Obstetrics and Gynecology	281	12.77	Clinical
Sibai et al. (1986a)	AJOG	276	7.67	Clinical
Rodgers et al. (1988)	AJOG	270	7.94	Clinical
Burton et al. (2009)	Placenta	269	20.69	Literature review

### Hot topics in the preeclampsia research

**FIGURE 4:**
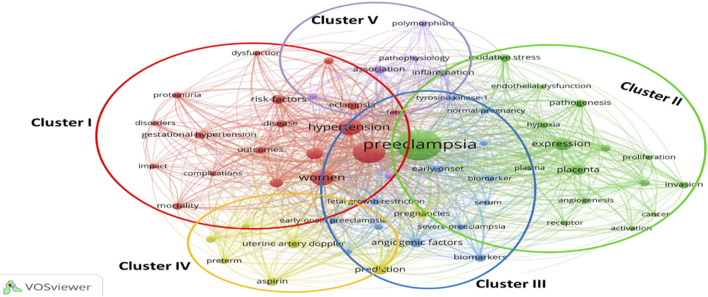
Co-occurrence map.

One of the best bibliometrics methods is keyword analysis. The evolution of a scientific domain is discovered using the co-occurrence method (Zhu et al., 2019). The co-occurrence technique is reliable and useful for selecting the most beneficial themes over time (Wang et al., 2018). Figure 4 shows the five clusters that were created from the 60 keywords (minimum keywords, 10 times): cluster one (n = 18 items), cluster two (n = 16), cluster three (n = 13), cluster four (n = 7), and cluster five (n = 6 things). The material for each cluster is displayed in Table 3.

**TABLE 3:** Classification of keywords based on clusters.

Cluster	Keywords
Cluster 1: 18 items Management of Preeclampsia	Cardiovascular-Disease, Complications, Disease, Disorders, Dysfunction, Eclampsia, Fetal, Gestational Hypertension, Hypertension, Hypertensive Disorders, Impact, Management, Mortality, Outcomes, Pregnancy, Proteinuria, Risk-Factors, Women
Cluster 2: 16 items Diagnosis of Preeclampsia	Activation, Angiogenesis, Cancer, Endothelial Dysfunction, Expression, Hypoxia, Invasion, Migration, Oxidative Stress, Pathogenesis, Placenta, Plasma, Preeclampsia, Proliferation, Receptor, Trophoblast
Cluster 3: 13 items The role of Biomarkers in Preeclampsia	Angiogenic Factors, Biomarker, Biomarkers, Early-Onset, Early-Onset Preeclampsia, Fetal, Growth Restriction, Late-Onset Preeclampsia, Normal-Pregnancy, Plgf, Serum, Severe, Preeclampsia, Soluble Endoglin, Tyrosine Kinase-1
Cluster 4: 7 items The role of Asprin in Preeclampsia	Aspirin, Growth Restriction, Low-Dose Aspirin, Prediction, Pregnancies, Preterm, Uterine Artery Doppler
Cluster 5: 6 items Preeclampsia and IUGR	Association, Blood-Pressure, Inflammation, Intrauterine Growth Restriction, Pathophysiology, Polymorphism

### Keywords trend

Topics that are expanding quickly in the field of preeclampsia were found by analyzing the frequency of keywords in subject categories. In Table 4, there are a few thematic groups with bursts and thorough details on the 25 thematic groups with the most powerful citation bursts. Preeclampsia, prostacyclin, endothelium, and thromboxane are identified by CiteSpace’s burst detection as the five topic areas with the most intense citation bursts. During a specific time, these topics were active research areas. Table 5 displays every colour section according to a timeline. A red line segment showing the beginning and finish of the burst duration is used to denote the length of a burst in a subject category. Pregnancy-induced hypertension, for instance, revealed a time of burst from 1992 to 2006 with a burst strength of 39.91 at the top of the list. The number of documents in the “pregnancy-induced hypertension” domain increased significantly between 1992 and 2006, indicating that preeclampsia research in this field was active at the time. Preeclampsia research has recently focused on topics including “cardiovascular disease,” “diagnosis,” “early onset,” “fetal growth restriction,” and “aspirin”.

**TABLE 4:** Top keywords with the strongest citation bursts.

Keywords	Year	Freq	Burst	Degree	Centrality	Strength	Begin	End	1992–2020
Pregnancy Induced Hypertension	1992	123	39.91	24	0.14	36.9	1992	2006	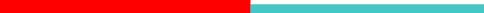
Preeclampsia	1992	64	33.83	15	0.02	31.25	1992	2003	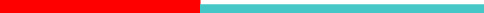
Prostacyclin	1992	42	22.15	19	0.06	20.35	1992	2003	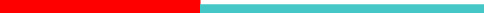
Endothelium	1992	24	12.63	12	0.01	11.41	1992	2004	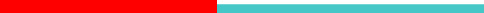
Thromboxane	1992	23	12.10	12	0.01	11.29	1992	2003	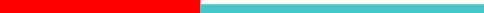
Endothelial Cell	1992	44	19.22	22	0.06	15.51	1993	2003	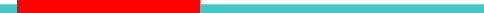
Rat	1992	27	-	17	0.04	12.51	1994	2000	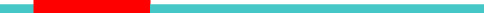
Nitric Oxide	1992	59	24.35	20	0.06	18.51	1995	2007	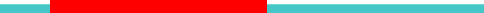
Activation	1992	74	21.53	21	0.06	18.81	1996	2009	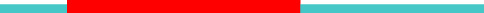
Lipid Peroxidation	1992	42	18.22	14	0.02	11.86	1996	2005	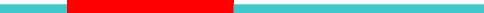
Insulin Resistance	1992	42	17.61	9	0.01	13.25	2005	2012	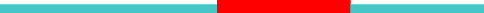
Soluble Endoglin	1992	66	17.93	12	0.01	15.37	2007	2014	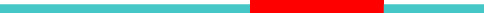
Gene Expression	1992	40	11.75	4	0.00	12.05	2009	2015	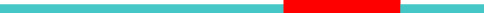
Circulating Angiogenic Factor	1992	39	12.59	14	0.02	13.44	2010	2014	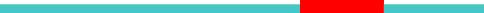
Placental Growth Factor	1992	100	14.61	15	0.02	12.95	2012	2020	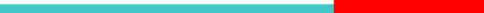
Angiogenic Factor	1992	170	16.46	18	0.03	19.15	2013	2017	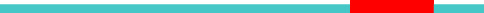
Biomarker	1992	103	16.65	15	0.02	18.06	2013	2020	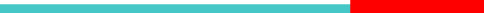
Prediction	1992	200	4.44	19	0.05	13.77	2013	2020	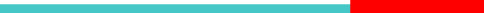
Uterine Artery Doppler	1992	136	11.33	14	0.01	11.08	2013	2016	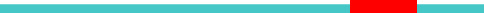
Prevention	1992	119	18.43	14	0.03	14.97	2014	2020	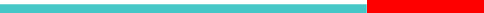
Cardiovascular Disease	1992	75	12.58	6	0.00	13.42	2014	2020	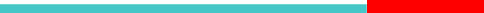
Diagnosis	1992	93	18.33	13	0.02	21.72	2015	2020	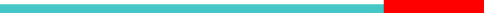
Early Onset	1992	40	16.56	2	0.00	14.1	2016	2020	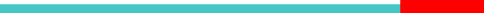
Fetal Growth Restriction	1992	63	14.97	6	0.00	13.5	2016	2020	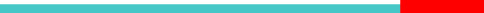
Aspirin	1992	54	16.81	13	0.02	14.1	2017	2020	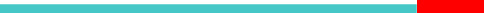

**TABLE 5:** The first five authors contribute to selected clusters.

Freq	Burst	Centrality	Source	References	Cluster	Carrot analysis
32	19.57	0.17	Lancet	Roberts and Redman (1993)	0	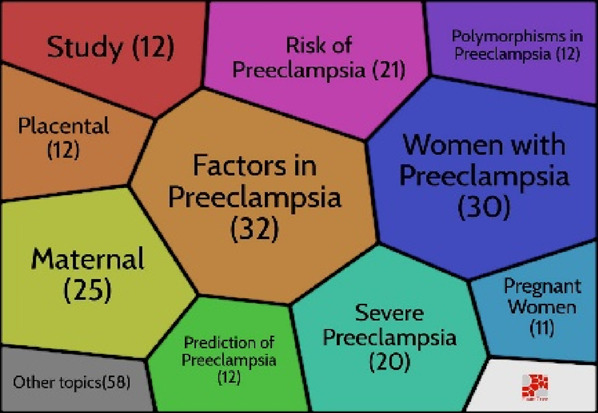
24	13.98	0.06	Am J Obstet Gynecol	Dekker and Sibai (1998)	0	
23	14.13	0.19	Am J Obstet Gynecol	Dekker et al. (1995)	0	
22	12.23	0.19	New Engl J Med	Kupferminc et al. (1999)	0	
17	10.20	0.00	New Engl J Med	Schobel et al. (1996)	0	
25	16.41	0.10	Am J Obstet Gynecol	Roberts et al. (1989)	1	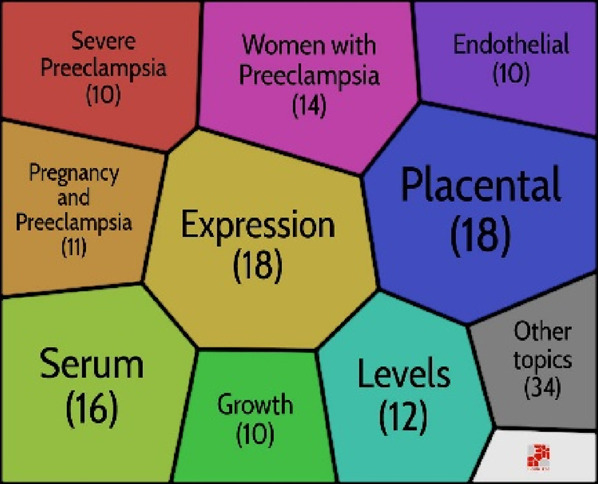
20	12.03	0.35	Am J Obstet Gynecol	Seligman et al. (1994)	1	
13	8.15	0.00	Clin Perinatol	Friedman et al. (1991)	1	
12	7.52	0.05	Am J Obstet Gynecol	Pinto et al. (1991)	1	
11	6.89	0.01	Am J Hypertens	Roberts et al. (1991)	1	
70	36.46	0.15	New Engl J Med	Levine et al. (2004)	2	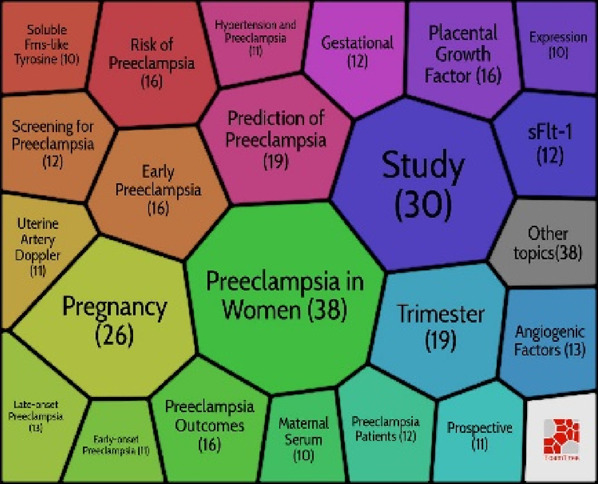
55	29.76	0.54	J Clin Invest	Maynard et al. (2003)	2	
52	31.12	0.03	Am J Obstet Gynecol	AuthorAnonymous (2000)	2	
40	23.39	0.59	Am J Obstet Gynecol	Redman et al. (1999)	2	
33	18.93	0.25	Lancet	Roberts & Cooper (2001)	2	
186	93.38	0.20	Obstet Gynecol	Roberts JM et al. (2013)	3	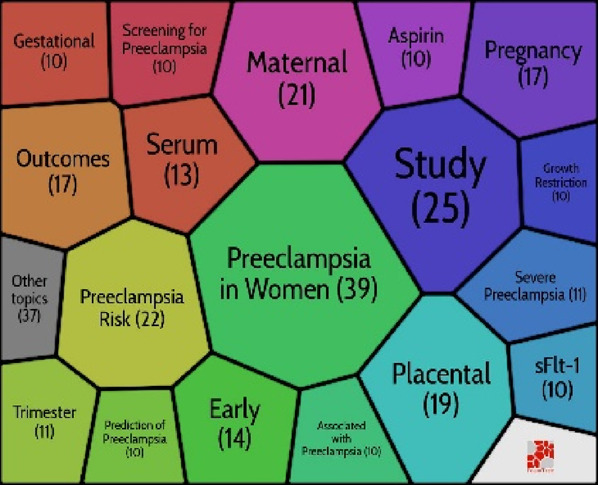
63	00.00	0.02	Pregnancy Hypertens	Tranquilli et al. (2014)	3	
62	29.47	0.09	New Engl J Med	Rolnik et al. (2018)	3	
51	23.56	0.05	Lancet	Mol et al. (2016)	3	
49	22.62	0.03	New Engl J Med	Zeisler et al. (2016)	3	
82	41.70	0.02	Lancet	Steegers et al. (2010)	4	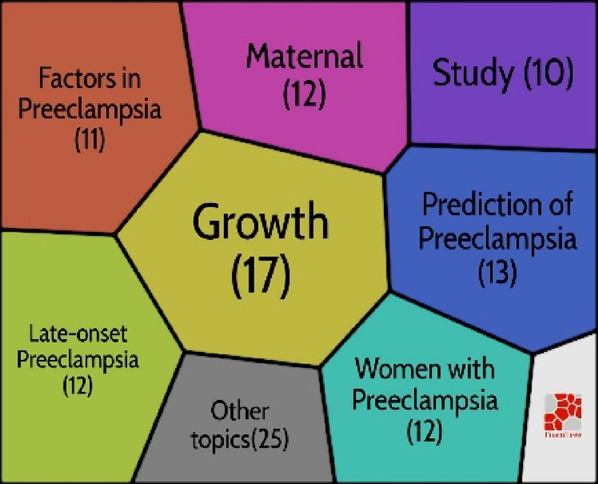
50	27.55	0.03	Semin Perinatol	Duley (2009)	4	
45	18.83	0.27	Am J Obstet Gynecol	Lisonkova & Joseph (2013)	4	
45	16.07	0.08	Fetal Diagn Ther	Akolekar et al. (2013)	4	
43	21.53	0.01	Obstet Gynecol	Bujold et al. (2010)	4	
73	39.03	0.00	Lancet	Sibai et al. (2005)	5	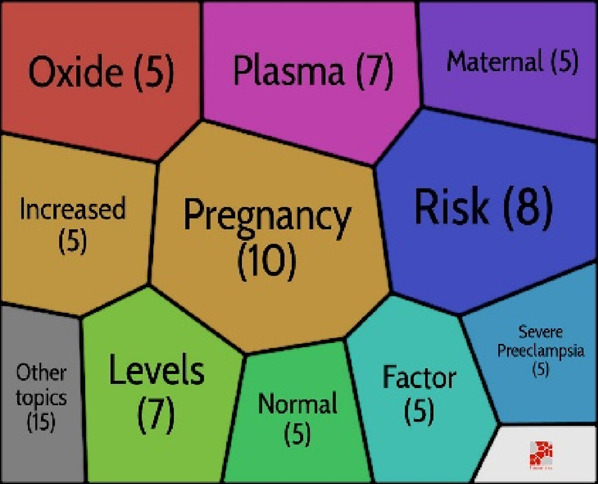
67	35.74	0.00	Science	Redman & Sargent (2005)	5	
62	31.23	0.23	New Engl J Med	Levine et al. (2006)	5	
47	23.53	0.20	Nat Med	Venkatesha et al. (2006)	5	
47	22.53	0.76	J Matern-Fetal Neo M	Romero et al. (2008)	5	

### Co-citation network

**FIGURE 5:**
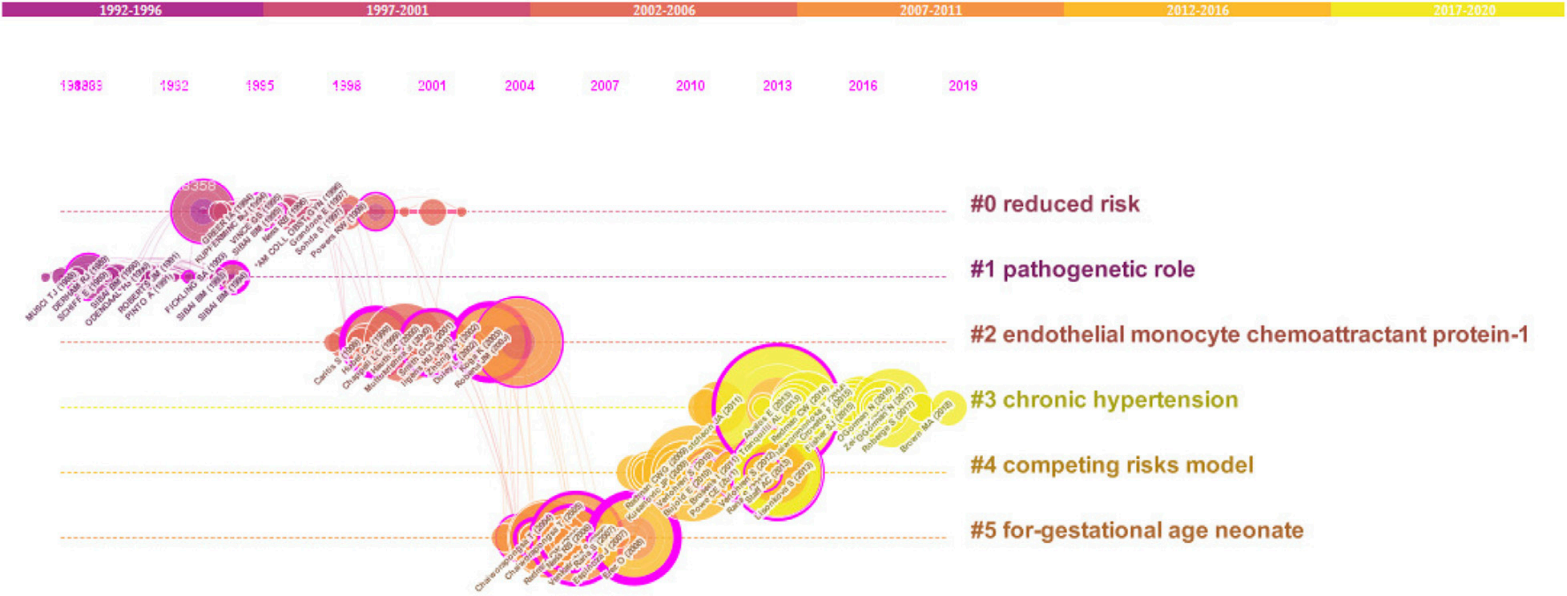
Co-citation grid of cited references.

Co-citation analysis is an effective technique that has gained widespread acceptance in several fields for accurately defining and visualising the intellectual structure of a study topic. In light of this, we decided to analyse the discipline’s structure using co-citation network analysis. Citespace software was used to investigate the preeclampsia discipline, which has its intellectual foundation in particular in the cited references. A network with 187 nodes and 598 edges was generated using document co-citation reference analysis in Citespace and the parameter settings: Slice length = 5; pruning: pathfinder; top N per slice: 30; top N%: 10%. (Figure 5). The LLR algorithm and title were used to cluster articles. Six clusters were ultimately procured, and Citespace gave them names automatically.

By using Carrot2, the clusters were deduced. To discover more about these clusters, the Carrot2 using the lingo algorithm was utilised on each cluster. The results showed that clusters #3 and #4 were the most recent clusters, while cluster #0 was the largest and oldest. Silhouette values of all clusters are bigger than 0.93. With 34 articles spread across 16 years, from 1985 to 2001, Cluster #0 is the largest cluster. Cluster #0 has a high degree of consistency, as evidenced by its Silhouette value of 0.936. Considering that Cluster #0 is the main cluster, its themes have been spread very widely. Carrot2 is capable of algorithmically managing the primary themes that are gleaned from the titles, keywords, and abstracts of cited documents (Table 5). The second-largest cluster, Cluster #1, contains 33 articles over the 10 years of 1985–1995 (Silhouette = 0.952). Cluster #3 is the most recent cluster (Silhouette = 0.992), and replicates significant events related to the risk of preeclampsia, placenta, sFlt-1, trimester, and growth restriction. Clusters #2, #4, and #5 all have silhouette values of 0.957, 0.949, and 0.935, respectively.

## Theme evolution: Strategic map in preeclampsia

Many keywords only occasionally appeared, therefore they undoubtedly had a significant impact on the key topic of preeclampsia. We involved the study’s title keywords. Using Bibliometrix^®^, a strategic map is created to examine the most important highlighted keywords in the preeclampsia field. The highly related keywords are gathered into clusters, and the themes are named using the most highly related terms (Wang et al., 2019). The strategic map is divided into four sections (Cobo et al., 2011), as indicated by Callon et al. (1991) including the basic themes (bottom right), emerging themes (bottom left), *motor themes* (top right), and highly developed themes (right-left). Motor themes have strong relationships and are quite determined. Themes that are thoroughly developed and isolated have strong internal connections but weak external links. *Themes that are developing or declining have a slight density and concentration, signifying weak interior and outer relations. Finally, basic and transversal themes, which are represented by topics with weak internal ties but large exterior links, have a high concentration and low density* (Cobo et al., 2011). The first five keywords and the number of times they appeared were as follows:

*Highly developed and isolated themes*: Hypertensive Disorders (249), Angiogenic Factors (149), Uterine Artery Doppler (122), Tyrosine Kinase-1 (86), and Soluble Endoglin (86).

*Motor themes*: Expression (355), Normal-Pregnancy (168), Plasma (164), Oxidative Stress (146), and Pathogenesis (142).

*Emerging or declining themes:* Pregnancy-induced hypertension (152), Pre-eclampsia (101), Elevated liver enzymes (84), Hemolysis (49), and Therapy (33).

*Basic and transversal themes:* Pregnancy (1084), Women (798), Hypertension (590), Risk (516), and Eclampsia (168).

## Discussion

In this study, by using bibliometric analysis, our research has explored and studied the trend of preeclampsia discipline between 1985 and 2020. The paper has valued the study and articles’ performance of authors, journals, organizations, and regions. To better recognize the configuration of the preeclampsia discipline, the authors advanced and studied co**-**citation, co-occurrence, strategic, and keywords maps. The chronological distribution displays three times in the publication movements in the preeclampsia discipline. The most cited document, ‘preeclampsia - an endothelial-cell disorder’ was published in the ‘AJOG’ (Roberts et al., 1989). The article’s from the ‘BJOJ’ was the second-most quoted piece of writing (Khong et al., 1986). Sacks article’s ‘AJOG’, which received the third-highest number of citations, was authored (Sacks et al., 1998). Sibai, Roberts, and Romero had the most preeclampsia-related documents among the authors. The United States, China, and England were the three most productive countries. The most effective institutions were ‘the University of Texas system’, ‘Harvard University’, and ‘PCSHE’. In terms of the number of articles, the overall number of citations, and productive organisations, the United States comes out on top. The ‘AJOG’, ‘Hypertension in Pregnancy’, and ‘Journal of Maternal-Fetal and Neonatal Medicine’ are the journals with the greatest impact.

**FIGURE 6:**
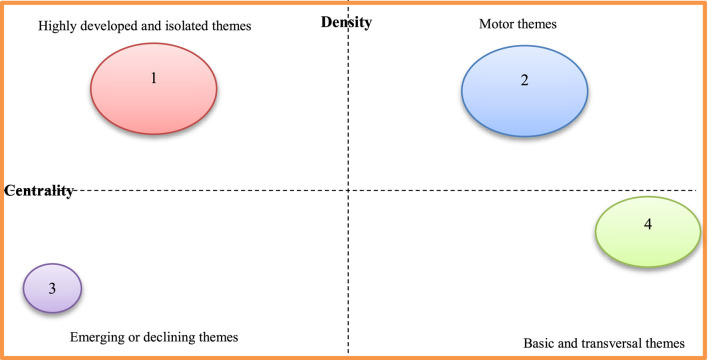
Strategic map in preeclampsia.

We discovered that preeclampsia is a developing but still challenging field of medicine, particularly obstetrics and gynaecology. The results of documents released between 1985 and 2020 revealed three stages in this field’s research: a seeding stage from 1985 to 1997, a rapid growth stage from 1997 to 2005, and a post-2005 stage (development stage). An examination of the keywords revealed a close connection to preeclampsia themes. As a result, the findings regarding the primary structuring of the mentioned sources were made clear by the keywords. This stability suggests that an appropriate study on this topic was developed along the progress process. Investigating the keywords revealed a network of keywords with five clusters in the study. According to this finding, the management, diagnosis, biomarker’s involvement in preeclampsia, aspirin’s role in preeclampsia, and the relationship between preeclampsia and IUGR are the primary areas of investigation in the preeclampsia field. The strategic keyword map’s findings revealed that a number of the keywords represent fundamental themes. The majority of motor topics have focused on expression, healthy pregnancy, plasma, oxidative stress, and pathophysiology, as shown in Figure 6 pregnancy-induced hypertension, preeclampsia prostacyclin, endothelium, and thromboxane were also the keywords with the greatest spikes in citations, but more recently, the focus of preeclampsia articles has shifted to include aspirin, cardiovascular disease, and early-onset, and fetal growth restriction. In other words, these areas have received more focus to evaluate the fetal and maternal impact of preeclampsia. Even though in previous years, these areas have received virtually little attention.

Six clusters were identified based on the results of co-citation, with clusters #3 and #4 being the most recent clusters and cluster #0 being the largest and oldest. Recently, the themes of chronic hypertension and conflicting risk models were studied. As a result, research on chronic hypertension and the competing risks model was focused on, along with studies on preeclampsia from lowered risks and the pathogenetic function. Researchers have been working on the chronic hypertension (Banala et al., 2020; Bramham et al., 2020; Hernández-Pacheco et al., 2020) as well as the competing risks model (Larroca et al., 2014; O’Gorman et al., 2016; Tsiakkas et al., 2016) in recent years.

## Conclusions and implications

The main cause of maternal and neonatal morbidity and mortality is preeclampsia (Liu T et al., 2019). Preeclampsia is a complication in pregnancy mainly described by the occurrence of hypertension, and proteinuria after ≥20 weeks of gestation, edema in pregnancy and accompanied by signs of damage to other organ systems (Moghaddami Tabrizi et al., 2001; Roberts et al., 2013). It is associated with a higher risk of impending death, cardiovascular disease, cerebrovascular illness, and persistent hypertension (Leslie & Briggs, 2016). Acute renal failure, liver rupture, pulmonary edema, and cerebrovascular accidents are among the preeclampsia complications that can cause maternal death (Adu-Bonsaffoh et al., 2013). Preeclampsia is managed by preventing seizures, reducing maternal hypertension, and having the fetus delivered on schedule (Yeo et al., 2014). Although the pathogenesis of this significant obstetric condition is still unknown (Chaiworapongsa et al., 2014; Burton et al., 2019; Phipps et al., 2019). Every study in our research is significant and deserving of examination by obstetricians and gynecologists in active practice. According to a bibliometric examination of highly referenced works in the field, the AJOG is the best leading journal in the preeclampsia discipline. More than 60% of these articles were published in the AJOG. Despite these constraints, many of the most popular articles in other fields are supported by the top-cited papers, co-citations, and co-occurrence maps. All scholars in the field of healthcare can use the bibliometric method to examine the trend in the chosen field.

## Contribution

This work makes a variety of contributions to theory and practice. The finding of this study was that the group of recently hot topics in this field that emerged from our research can serve as the foundation for future research. This study makes a significant contribution for researchers involved in preeclampsia because not only we shape, structure, and recognize the key universities, journals, documents and authors to be taken into consideration when conducting future research on preeclampsia but also to design a new trend in this field.

## Strengths and limitations

This study has several restrictions. The WoS database is the only one that may be used for this research. Although it is the source with the most documentation, the information it contains is limited. This database does not index certain journals. As a result, other sources like PubMed, Google Scholar, and Scopus are available for use by other researchers. The second restriction is that an author could only have a minimum of 10 documents and a maximum of 10 keyword phrases. Third, this bibliometric analysis only considers publications in the fields of obstetrics and gynecology; other researchers may look into the fields of reproductive biology, physiology, general internal medicine, developmental biology, and biochemistry molecular biology. This study’s greatest asset was the thorough review of articles from the very beginning to the present.

## References

[B1] Adu-Bonsaffoh K. Oppong S. A. Binlinla G. Samuel O. A. (2013). Maternal deaths attributable to hypertensive disorders in a tertiary hospital in Ghana. Int. J. Gynecol. Obstetrics 123 (2), 110–113. 10.1016/j.ijgo.2013.05.017 23969337

[B2] Akolekar R. Syngelaki A. Poon L. Wright D. Nicolaides K. H. (2013). Competing risks model in early screening for preeclampsia by biophysical and biochemical markers. Fetal Diagnosis Ther. 33 (1), 8–15. 10.1159/000341264 22906914

[B3] Alarcon-Ruiz C. A. Diaz-Barrera M. E. Vera-Monge V. A. Alva-Diaz C. Metcalf T. (2019). A bibliometric analysis of the Latin American research on stroke 2003–2017. World Neurosurg. 129, e545–e554. 10.1016/j.wneu.2019.05.212 31152886

[B4] Allaire A. D. Ballenger K. A. Wells S. R. McMahon M. J. Lessey B. A. (2000). Placental apoptosis in preeclampsia. Obstetrics Gynecol. 96 (2), 271–276. 10.1016/S0029-7844(00)00895-4 10908776

[B5] AuthorAnonymous (2000). Report of the national high blood pressure education program working group on high blood pressure in pregnancy. Am. J. Obstetrics Gynecol. 183 (1), s1–s22. 10.1067/mob.2000.107928 10920346

[B6] Banala C. Moreno S. Cruz Y. Boelig R. C. Saccone G. Berghella V. (2020). Mothers with long QT syndrome are at increased risk for fetal death: Findings from a multicenter international study. Am. J. Obstetrics Gynecol. 223 (3), e1–e263. 10.1016/j.ajog.2019.09.004 31520628

[B7] Bramham K. Villa P. M. Joslin J. R. Laivuori H. Hämäläinen E. Kajantie E. (2020). Predisposition to superimposed preeclampsia in women with chronic hypertension: Endothelial, renal, cardiac, and placental factors in a prospective longitudinal cohort. Hypertens. Pregnancy 39 (3), 326–335. 10.1080/10641955.2020.1769643 32479208

[B8] Broadus R. N. (1987). Toward a definition of “bibliometrics. Scientometrics 12 (5), 373–379. 10.1007/BF02016680

[B9] Bujold E. Roberge S. Lacasse Y. Bureau M. Audibert F. Marcoux S. (2010). Prevention of preeclampsia and intrauterine growth restriction with aspirin started in early pregnancy A meta-analysis. Obstetrics Gynecol. 116, 402–414. 10.1097/AOG.0b013e3181e9322a 20664402

[B10] Burton G. J. Redman C. W. Roberts J. M. Moffett A. (2019). Pre-eclampsia: Pathophysiology and clinical implications. Bmj 366, l2381. 10.1136/bmj.l2381 31307997

[B11] Burton G. J. Yung H.-W. Cindrova-Davies T. Charnock-Jones D. S. (2009). Placental endoplasmic reticulum stress and oxidative stress in the pathophysiology of unexplained intrauterine growth restriction and early onset preeclampsia. Placenta 30, S43–S48. 10.1016/j.placenta.2008.11.003 19081132 PMC2684656

[B12] Butt I. Iqbal T. Zohaib S. (2019). Healthcare marketing: A review of the literature based on citation analysis. Health Mark. Q. 0 (0), 271–290. 10.1080/07359683.2019.1680120 31646959

[B13] Callon M. Courtial J. P. Laville F. (1991). Co-word analysis as a tool for describing the network of interactions between basic and technological research: The case of polymer chemsitry. Scientometrics 22 (1), 155–205. 10.1007/BF02019280

[B14] Chaiworapongsa T. Chaemsaithong P. Yeo L. Romero R. Services H. (2014). Pre-eclampsia part 1: Current understanding of its pathophysiology. Nat. Rev. Nephrol. 10 (8), 466–480. 10.1038/nrneph.2014.102 25003615 PMC5893150

[B15] Chen C. (2004). Searching for intellectual turning points: Progressive knowledge domain visualization. Proc. Natl. Acad. Sci. U. S. A. 101 (1), 5303–5310. 10.1073/pnas.0307513100 14724295 PMC387312

[B16] Cobo M. J. López-Herrera A. G. Herrera-Viedma E. Herrera F. (2011). An approach for detecting, quantifying, and visualizing the evolution of a research field: A practical application to the fuzzy sets theory field. J. Inf. 5 (1), 146–166. 10.1016/j.joi.2010.10.002

[B17] De Bellis N. (2009). Bibliometrics and citation analysis: From the science citation index to cybermetrics. Lanham, MD: scarecrow press.

[B18] Dekker G. A. Devries J. I. P. Doelitzsch P. M. Huijgens P. C. Vonblomberg B. M. E. Jakobs C. (1995). Underlying disorders associated with severe early-onset preeclampsia. Am. J. Obstetrics Gynecol. 173 (4), 1042–1048. 10.1016/0002-9378(95)91324-6 7485291

[B19] Dekker G. A. Sibai B. M. (1998). Etiology and pathogenesis of preeclampsia: Current concepts. Am. J. Obstetrics Gynecol. 179 (5), 1359–1375. 10.1016/s0002-9378(98)70160-7 9822529

[B20] Dias G. P. (2019). Fifteen years of e-government research in ibero-America: A bibliometric analysis. Gov. Inf. Q. 36 (3), 400–411. 10.1016/j.giq.2019.05.008

[B21] Duley L. (2009). The global impact of pre-eclampsia and eclampsia. Seminars Perinatology 33 (3), 130–137. 10.1053/j.semperi.2009.02.010 19464502

[B22] Friedman S. A. Taylor R. N. Roberts J. M. (1991). Pathophysiology of preeclampsia. Clin. Perinatology 18 (4), 661–682. 10.1016/S0095-5108(18)30490-1 1764879

[B23] Gilstrap L. C. Ramin S. M. (2002). Diagnosis and management of preeclampsia and eclampsia. ACOG Pract. Bull. 33, 1–9.

[B24] Gupta A. Kennedy B. Meriwether K. V. Francis S. L. Cardenas-Trowers O. Stewart J. R. (2019). Citation classics: The 100 most cited articles in urogynecology. Int. Urogynecology J. 31, 249–266. 10.1007/s00192-019-04021-9 31309245

[B25] Hernández-Pacheco J. A. Rosales-Zamudio C. I. Borboa-Olivares H. Espejel-Núñez A. Parra-Hernández S. Estrada-Gutiérrez G. (2020). The sFlt-1/PlGF ratio as a triage tool to identify superimposed preeclampsia in women with chronic hypertension in emergency rooms. Pregnancy Hypertens. 21, 38–42. 10.1016/j.preghy.2020.04.014 32388017

[B26] Ishihara N. Matsuo H. Murakoshi H. Laoag-Fernandez J. B. Samoto T. Maruo T. (2002). Increased apoptosis in the syncytiotrophoblast in human term placentas complicated by either preeclampsia or intrauterine growth retardation. Am. J. Obstetrics Gynecol. 186 (1), 158–166. 10.1067/mob.2002.119176 11810103

[B27] Jeung C.-W. Yoon H. J. Park S. Jo S. J. (2011). The contributions of human resource development research across disciplines: A citation and content analysis. Hum. Resour. Dev. Q. 22 (1), 87–109. 10.1002/hrdq.20062

[B28] Karydakis P. Giakoumettis D. Themistocleous M. (2019). The 100 most cited papers about pediatric traumatic brain injury: A bibliometric analysis. Ir. J. Med. Sci. 189, 315–325. 10.1007/s11845-019-02085-6 31418153

[B29] Kazemi Aski S. Akbari R. Hantoushzadeh S. Ghotbizadeh F. (2020). A bibliometric analysis of Intrauterine Growth Restriction research. Placenta 95, 106–120. 10.1016/j.placenta.2020.03.010 32452397

[B30] Khong T. Y. De Wolf F. Robertson W. B. Brosens I. (1986). Inadequate maternal vascular response to placentation in pregnancies complicated by pre‐eclampsia and by small‐for‐gestational age infants. BJOG Int. J. Obstetrics Gynaecol. 93 (10), 1049–1059. 10.1111/j.1471-0528.1986.tb07830.x 3790464

[B31] Kupferminc M. J. Eldor A. Steinman N. Many A. Bar-Am A. Jaffa A. (1999). Increased frequency of genetic thrombophilia in women with complications of pregnancy. N. Engl. J. Med. 340 (1), 9–13. 10.1056/NEJM199901073400102 9878639

[B32] Kurki T. Hiilesmaa V. Raitasalo R. Mattila H. Ylikorkala O. (2000). Depression and anxiety in early pregnancy and risk for preeclampsia. Obstetrics Gynecol. 95 (4), 487–490. 10.1016/S0029-7844(99)00602-X 10725477

[B33] Larroca S. G.-T. Tayyar A. Poon L. C. Wright D. Nicolaides K. H. (2014). Competing risks model in screening for preeclampsia by biophysical and biochemical markers at 30-33 Weeks’ gestation. FETAL DIAGNOSIS Ther. 36 (1), 9–17. 10.1159/000362518 24902880

[B34] Lee A. Ramstein J. Cohen A. J. Agochukwu-Mmonu N. Patino G. Breyer B. N. (2019). The top 100 cited articles in urethral reconstruction. Urology 135, 139–145. 10.1016/j.urology.2019.08.052 31586471

[B35] Leslie M. S. Briggs L. A. (2016). Preeclampsia and the risk of future vascular disease and mortality: A review. J. Midwifery & Women’s Health 61 (3), 315–324. 10.1111/jmwh.12469 27155218

[B36] Levine R. J. Lam C. Qian C. Yu K. F. Maynard S. E. Sachs B. P. (2006). Soluble endoglin and other circulating antiangiogenic factors in preeclampsia. N. Engl. J. Med. 355 (10), 992–1005. 10.1056/NEJMoa055352 16957146

[B37] Levine R. J. Maynard S. E. Qian C. Lim K.-H. England L. J. Yu K. F. (2004). Circulating angiogenic factors and the risk of preeclampsia. N. Engl. J. Med. 350 (7), 672–683. 10.1056/NEJMoa031884 14764923

[B38] Lisonkova S. Joseph K. S. (2013). Incidence of preeclampsia: Risk factors and outcomes associated with early-versus late-onset disease. Am. J. Obstetrics Gynecol. 209 (6), e1–e544. 10.1016/j.ajog.2013.08.019 23973398

[B39] Liu T. Zhang M. Guallar E. Wang G. Hong X. Wang X. (2019). Trace minerals, heavy metals, and preeclampsia: Findings from the boston birth cohort. J. Am. Heart Assoc. 8 (16), e012436. 10.1161/JAHA.119.012436 31426704 PMC6759885

[B40] Liu W. Zhang Y. Wu L. Yang X. Shi L. (2019). Characteristics and trends of oral leukoplakia research: A bibliometric study of the 100 most cited articles. Medicine 98 (27), e16293. 10.1097/MD.0000000000016293 31277163 PMC6635245

[B41] Liu X. Zhang J. Guo C. (2013). Full-text citation analysis: A new method to enhance scholarly networks. J. Am. Soc. Inf. Sci. Technol. 64, 1852–1863. 10.1002/asi.22883

[B42] MacKay A. P. Berg C. J. Atrash H. K. (2001). Pregnancy-related mortality from preeclampsia and eclampsia. Obstetrics Gynecol. 97 (4), 533–538. 10.1016/S0029-7844(00)01223-0 11275024

[B43] Maynard S. E. Min J.-Y. Merchan J. Lim K.-H. Li J. Mondal S. (2003). Excess placental soluble fms-like tyrosine kinase 1 (sFlt1) may contribute to endothelial dysfunction, hypertension, and proteinuria in preeclampsia. J. Clin. Investigation 111 (5), 649–658. 10.1172/JCI17189 PMC151901 12618519

[B44] Moghaddami Tabrizi N. Eazadi Mood N. Tahmasbi M. (2001). Midtrimester serum B-subunit human chorionic gonadotropin levels and the subsequent development of preeclampsia. Tehran Univ. Med. J. 59 (4), 63–66. http://tumj.tums.ac.ir/article-1-1312-fa.html.

[B45] Mol B. W. J. Roberts C. T. Thangaratinam S. Magee L. A. de Groot C. J. M. Hofmeyr G. J. (2016). Pre-eclampsia. Lancet 387 (10022), 999–1011. 10.1016/S0140-6736(15)00070-7 26342729

[B46] Ness R. B. Roberts J. M. (1996). Heterogeneous causes constituting the single syndrome of preeclampsia: A hypothesis and its implications. Am. J. Obstetrics Gynecol. 175 (5), 1365–1370. 10.1016/S0002-9378(96)70056-X 8942516

[B47] Ødegård R. A. Vatten L. J. Nilsen S. T. Salvesen K. Å. Austgulen R. (2000). Preeclampsia and fetal growth. Obstetrics Gynecol. 96 (6), 950–955. 10.1016/S0029-7844(00)01040-1 11084184

[B48] O’Gorman N. Wright D. Syngelaki A. Akolekar R. Wright A. Poon L. C. (2016). Competing risks model in screening for preeclampsia by maternal factors and biomarkers at 11-13 weeks gestation. Am. J. Obstetrics Gynecol. 214 (1), e1–e103. 10.1016/j.ajog.2015.08.034 26297382

[B49] Phipps E. A. Thadhani R. Benzing T. Karumanchi S. A. (2019). Pre-eclampsia: Pathogenesis, novel diagnostics and therapies. Nat. Rev. Nephrol. 15 (5), 275–289. 10.1038/s41581-019-0119-6 30792480 PMC6472952

[B50] Phipps E. Prasanna D. Brima W. Jim B. (2016). Preeclampsia: Updates in pathogenesis, definitions, and guidelines. Clin. J. Am. Soc. Nephrol. 11 (6), 1102–1113. 10.2215/CJN.12081115 27094609 PMC4891761

[B51] Pineles B. L. Romero R. Montenegro D. Tarca A. L. Han Y. M. Kim Y. M. (2007). Distinct subsets of microRNAs are expressed differentially in the human placentas of patients with preeclampsia. Am. J. Obstetrics Gynecol. 196 (3), 261–e6. 10.1016/j.ajog.2007.01.008 17346547

[B52] Pinto A. Sorrentino R. Sorrentino P. Guerritore T. Miranda L. Biondi A. (1991). Endothelial-derived relaxing factor released by endothelial cells of human umbilical vessels and its impairment in pregnancy-induced hypertension. Am. J. Obstetrics Gynecol. 164 (2), 507–513. 10.1016/S0002-9378(11)80010-4 1992693

[B53] Pritchard A. (1969). Statistical bibliography or bibliometrics. J. Documentation 25 (4), 348–349.

[B54] Redman C. W. G. Sacks G. P. Sargent I. L. (1999). Preeclampsia: An excessive maternal inflammatory response to pregnancy. Am. J. Obstetrics Gynecol. 180 (2), 499–506. 10.1016/s0002-9378(99)70239-5 9988826

[B55] Redman C. W. Sargent I. L. (2005). Latest advances in understanding preeclampsia. Science 308 (5728), 1592–1594. 10.1126/science.1111726 15947178

[B56] Roberts J. M. Pa A. Bakris G. Barton J. R. Im B. (2013). Hypertension in pregnancy. Report of the American college of obstetricians and gynecologists’ task force on hypertension in pregnancy. Obstetrics Gynecol. 122 (5), 1122–1131. 10.1097/01.AOG.0000437382.03963.88 24150027

[B57] Roberts J. M. Cooper D. W. (2001). Pathogenesis and genetics of pre-eclampsia. Lancet 357 (9249), 53–56. 10.1016/s0140-6736(00)03577-7 11197372

[B58] Roberts J. M. Hubel C. A. (2009). The two stage model of preeclampsia: Variations on the theme. PLACENTA 30, S32–S37. 10.1016/j.placenta.2008.11.009 19070896 PMC2680383

[B59] Roberts J. M. Redman C. W. G. (1993). Pre-eclampsia: More than pregnancy-induced hypertension. Lancet 341 (8858), 1447–1451. 10.1016/0140-6736(93)90889-O 8099148

[B60] Roberts J. M. Taylor R. N. Goldfien A. (1991). Clinical and biochemical evidence of endothelial cell dysfunction in the pregnancy syndrome preeclampsia. Am. J. Hypertens. 4 (8), 700–708. 10.1093/ajh/4.8.700 1930853

[B61] Roberts J. M. Taylor R. N. Musci T. J. Rodgers G. M. Hubel C. A. McLaughlin M. K. (1989). Preeclampsia: An endothelial cell disorder. Am. J. Obstetrics Gynecol. 161 (5), 1200–1204. 10.1016/0002-9378(89)90665-0 2589440

[B62] Rodgers G. M. Taylor R. N. Roberts J. M. (1988). Preeclampsia is associated with a serum factor CYTO-toxic to human-endothelial cells. Am. J. Obstetrics Gynecol. 159 (4), 908–914. 10.1016/S0002-9378(88)80169-8 3177546

[B63] Rolnik D. L. Wright D. Poon L. C. O’Gorman N. Syngelaki A. de Paco Matallana C. (2018). Aspirin versus placebo in pregnancies at high risk for preterm preeclampsia. Obstetrical Gynecol. Surv. 73 (1), 11–12. 10.1097/01.ogx.0000528015.09400.25 28657417

[B64] Romero R. Nien J. K. Espinoza J. Todem D. Fu W. Chung H. (2008). A longitudinal study of angiogenic (placental growth factor) and anti-angiogenic (soluble endoglin and soluble vascular endothelial growth factor receptor-1) factors in normal pregnancy and patients destined to develop preeclampsia and deliver a small for gestational age neonate. J. Maternal-Fetal Neonatal Med. 21 (1), 9–23. 10.1080/14767050701830480 PMC2587364 18175241

[B65] Sacks G. P. Studena K. Sargent I. L. Redman C. W. G. (1998). Normal pregnancy and preeclampsia both produce inflammatory changes in peripheral blood leukocytes akin to those of sepsis. Am. J. Obstetrics Gynecol. 179 (1), 80–86. 10.1016/s0002-9378(98)70254-6 9704769

[B66] Saftlas A. F. Olson D. R. Franks A. L. Atrash H. K. Pokras R. (1990). Epidemiology of preeclampsia and eclampsia in the united-states, 1979-1986. Am. J. Obstetrics Gynecol. 163 (2), 460–465. 10.1016/0002-9378(90)91176-D 2386132

[B67] Schobel H. P. Fischer T. Heuszer K. Geiger H. Schmieder R. E. (1996). Preeclampsia - a state of sympathetic overactivity. N. Engl. J. Med. 335 (20), 1480–1485. 10.1056/NEJM199611143352002 8890098

[B68] Seligman S. P. Buyon J. P. Clancy R. M. Young B. K. Abramson S. B. (1994). The role of nitric-oxide in the pathogenesis of preeclampsia. Am. J. Obstetrics Gynecol. 171 (4), 944–948. 10.1016/S0002-9378(94)70064-8 7943106

[B69] Sibai B. Dekker G. Kupferminc M. (2005). Pre-eclampsia. Lancet 365 (9461), 785–799. 10.1016/S0140-6736(05)17987-2 15733721

[B70] Sibai B. M. Elnazer A. Gonzalezruiz A. el-Nazer A. GonzAlez-Ruiz A. (1986a). Severe preeclampsia-eclampsia in young primigravid women - subsequent pregnancy outcome and remote prognosis. Am. J. Of Obstetrics And Gynecol. 155 (5), 1011–1016. 10.1016/0002-9378(86)90336-4 3777042

[B71] Sibai B. M. Ewell M. Levine R. J. Klebanoff M. A. Esterlitz J. Catalano P. M. (1997). Risk factors associated with preeclampsia in healthy nulliparous women. The Calcium for Preeclampsia Prevention (CPEP) Study Group. Am. J. Of Obstetrics And Gynecol. 177 (5), 1003–1010. 10.1016/S0002-9378(97)70004-8 9396883

[B72] Sibai B. M. Gordon T. Thom E. Caritis S. N. Klebanoff M. Mcnellis D. (1995). Risk factors for preeclampsia in healthy nulliparous women: A prospective multicenter study. The national institute of child health and human development network of maternal-fetal medicine units. Am. J. Of Obstetrics And Gynecol. 172 (21), 642–648. 10.1016/0002-9378(95)90586-3 7856699

[B73] Sibai B. M. Mercer B. M. Schiff E. Friedman S. A. (1994). Aggressive versus expectant management of severe preeclampsia at 28 to 32 weeks gestation - a randomized controlled trial. Am. J. Of Obstetrics And Gynecol. 171 (3), 818–822. 10.1016/0002-9378(94)90104-X 8092235

[B74] Sibai B. M. Taslimi M. M. El-Nazer A. Amon E. Mabie B. C. Ryan G. M. (1986b). Maternal-perinatal outcome associated with the syndrome of hemolysis, elevated liver enzymes, and low platelets in severe preeclampsia-eclampsia. Am. J. Obstetrics Gynecol. 155 (3), 501–509. 10.1016/0002-9378(86)90266-8 3529964

[B75] States U. Shah R. Kashkoush J. Kashkoush A. Patel T. (2019). Analysis of the top 100 most influential papers in benign prostatic hyperplasia. Can. Urological Assoc. J. = J. de l’Association Des Urologues Du Can. 14. 10.5489/cuaj.5831 31658011

[B76] Steegers E. A. P. Von Dadelszen P. Duvekot J. J. Pijnenborg R. (2010). Pre-eclampsia. Lancet 376 (9741), 631–644. 10.1016/S0140-6736(10)60279-6 20598363

[B77] Tran B. X. Latkin C. A. Vu G. T. Nguyen H. L. T. Nghiem S. Tan M.-X. (2019). The current research landscape of the application of artificial intelligence in managing cerebrovascular and heart diseases: A bibliometric and content analysis. Int. J. Environ. Res. Public Health 16 (15), 2699. 10.3390/ijerph16152699 31362340 PMC6696240

[B78] Tranquilli A. Dekker G. Magee L. Roberts J. Sibai B. M. Steyn W. (2014). The classification, diagnosis and management of the hypertensive disorders of pregnancy: A revised statement from the ISSHP. Pregnancy Hypertens. 4 (2), 97–104. 10.1016/j.preghy.2014.02.001 26104417

[B79] Tsiakkas A. Saiid Y. Wright A. Wright D. Nicolaides K. H. (2016). Competing risks model in screening for preeclampsia by maternal factors and biomarkers at 30-34 weeks’ gestation. Am. J. Obstetrics Gynecol. 215 (1), e1–e87. 10.1016/j.ajog.2016.02.016 26875953

[B80] Van Eck N. J. Waltman L. (2009). Software survey: VOSviewer, a computer program for bibliometric mapping. Scientometrics 84 (2), 523–538. 10.1007/s11192-009-0146-3 20585380 PMC2883932

[B81] Venkatesha S. Toporsian M. Lam C. Hanai J. Mammoto T. Kim Y. M. (2006). Soluble endoglin contributes to the pathogenesis of preeclampsia. Nat. Med. 12 (6), 642–649. 10.1038/nm1429 16751767

[B82] Villar J. Carroli G. Wojdyla D. Abalos E. Giordano D. Ba’aqeel H. (2006). Preeclampsia, gestational hypertension and intrauterine growth restriction, related or independent conditions? Am. J. Of Obstetrics And Gynecol. 194 (4), 921–931. 10.1016/j.ajog.2005.10.813 16580277

[B83] Vince G. S. Starkey P. M. Austgulen R. Kwiatkowski D. Redman C. W. G. (1995). Interleukin-6, tumour necrosis factor and soluble tumour necrosis factor receptors in women with pre-eclampsia. Br. J. Of Obstetrics And Gynaecol. 102 (1), 20–25. 10.1111/j.1471-0528.1995.tb09020.x 7833306

[B84] von Dadelszen P. Magee L. A. Roberts J. M. (2003). Subclassification of preeclampsia. Hypertens. Pregnancy 22 (2), 143–148. 10.1081/PRG-120021060 12908998

[B85] Walsh S. W. (1985). Preeclampsia: An imbalance in placental prostacyclin and thromboxane production. Am. J. Obstetrics Gynecol. 152 (3), 335–340. 10.1016/s0002-9378(85)80223-4 3923838

[B86] Wang C. Lim M. K. Zhao L. Tseng M.-L. Chien C.-F. Lev B. (2019). The evolution of omega-the international journal of management science over the past 40 years: A bibliometric overview. Omega 93, 102098. 10.1016/j.omega.2019.08.005

[B87] Wang P. Zhu F. W. Song H. Y. Hou J. H. Zhang J. L. (2018). Visualizing the academic discipline of knowledge management. Sustain. Switz. 10 (3), 682. 10.3390/su10030682

[B88] Witlin A. Sibai B. (1998). Magnesium sulfate therapy in preeclampsia and eclampsia. Obstetrics Gynecol. 92 (5), 883–889. 10.1016/s0029-7844(98)00277-4 9794688

[B89] Xu J. Zhang Y. Wu Y. Wang J. Dong X. Xu H. (2015). Citation sentiment analysis in clinical trial papers. AMIA Annu. Symp. Proc. 2015, 1334–1341. https://www.ncbi.nlm.nih.gov/pubmed/26958274. 26958274 PMC4765697

[B90] Yadava S. M. Patrick H. S. Ananth C. V. Rosen T. Brandt J. S. (2019). Top-cited articles in the journal: A bibliometric analysis. Am. J. Obstetrics Gynecol. 220 (1), 12–25. 10.1016/j.ajog.2018.11.1091 30452887

[B91] Yallampalli C. Garfield R. E. (1993). Inhibition of nitric-oxide synthesis in rats during pregnancy produces signs similar to those of preeclampsia. Am. J. Of Obstetrics And Gynecol. 169 (5), 1316–1320. 10.1016/0002-9378(93)90299-X 8238200

[B92] Yeo L. Romero R. Sci D. M. Romero R. (2014). Pre-eclampsia part 2: Prediction, prevention and management. Nat. Rev. Nephrol. 10 (9), 531–540. 10.1038/nrneph.2014.103 25003612 PMC5898797

[B93] Zeisler H. Llurba E. Chantraine F. Vatish M. Staff A. C. Sennström M. (2016). Predictive value of the sFlt-1: PlGF ratio in women with suspected preeclampsia. N. Engl. J. Med. 374, 13–22. 10.1056/NEJMoa1414838 26735990

[B94] Zhu J. Song L. J. Zhu L. Johnson R. E. (2019). Visualizing the landscape and evolution of leadership research. Leadersh. Q. 30 (2), 215–232. 10.1016/j.leaqua.2018.06.003

